# Tailoring the HHx monomer content of P(HB-*co*-HHx) by flexible substrate compositions: scale-up from deep-well-plates to laboratory bioreactor cultivations

**DOI:** 10.3389/fbioe.2023.1081072

**Published:** 2023-05-02

**Authors:** Lara Santolin, Isabel Thiele, Peter Neubauer, Sebastian L. Riedel

**Affiliations:** ^1^ Technische Universität Berlin, Institute of Biotechnology, Chair of Bioprocess Engineering, Berlin, Germany; ^2^ Berliner Hochschule für Technik, Department VIII – Mechanical Engineering, Event Technology and Process Engineering, Laboratory of Environmental and Bioprocess Engineering, Berlin, Germany

**Keywords:** bioplastic, PHA, polyhydroxyalkanoate, poly(hydroxybutyrate-*co*-hydroxyhexanoate), *Ralstonia eutropha*, substrate-flexible, biodegradable, scale-up

## Abstract

The enhanced material properties exhibited by the microbially synthetized polyhydroxyalkanoate (PHA) copolymer poly(hydroxybutyrate-*co*-hydroxyhexanoate) [P(HB-*co*-HHx)] evidence that this naturally biodegrading biopolymer could replace various functionalities of established petrochemical plastics. In fact, the thermal processability, toughness and degradation rate of P(HB-*co*-HHx) can be tuned by modulating its HHx molar content enabling to manufacture polymers à-la-carte. We have developed a simple batch strategy to precisely control the HHx content of P(HB-*co*-HHx) to obtain tailor-made PHAs with defined properties. By adjusting the ratio of fructose to canola oil as substrates for the cultivation of recombinant *Ralstonia eutropha* Re2058/pCB113, the molar fraction of HHx in P(HB-*co*-HHx) could be adjusted within a range of 2–17 mol% without compromising polymer yields. The chosen strategy proved to be robust from the mL-scale in deep-well-plates to 1-L batch bioreactor cultivations.

## 1 Introduction

From the poles to the deep ocean basins, plastic pollution has reached every remote corner of our planet. While marine and freshwater ecosystems are threatened by up to 23 million tons of plastics entering the oceans each year, the petrochemical plastics industry is thriving and plastic production could reach over 600 million tons produced by 2030 (Borelle et al., 2020; MacLeod et al., 2021). To counter this threat, legislations must be enacted to curb plastic waste generation and promote the transition to more environmentally friendly yet competitive materials (Gutschmann et al., 2022a).

A key role in this group is played by polyhydroxyalkanoates (PHAs), microbially produced bioplastics that are stored by various microorganisms from various carbon sources as energy and carbon storage compounds. The most common type of PHA is the homopolymer polyhydroxybutyrate (PHB) that was shown to degrade in various environments to CO_2_ and water (Narancic and O’Connor, 2019). However, this thermoplastic is very crystalline and has a high melting point (175°C) which is close to its degradation temperature, making the processing window too small and limiting its practical application (Noda et al., 2005). To be of a real value as replacement for commodity plastics, copolymerization of HB units with longer chain length monomers is often targeted, which reduces the melting temperature and weakens the crystalline structure by steric hindrance (Grigore et al., 2019). Poly(hydroxybutyrate-*co*-hydroxyhexanoate) (P(HB-*co*-HHx)) is one of such copolymers and, while biodegradability of PHAs in each environment is strongly affected by the monomer composition and post-processing, this copolymer also shows full biodegradability in soil and seawater (Narancic and O’Connor, 2019; Riedel and Brigham, 2020; Amasawa et al., 2021). Many efforts have been made to genetically modify the PHA operon of the model organism for PHA production, *Ralstonia eutropha,* in order to produce P(HB-*co*-HHx) from related carbon sources, where HHx precursors are generated from intermediates of the ß-oxidation of fatty acids, like palm oil (Budde, et al., 2011b; Riedel et al., 2014), as well as from unrelated carbon sources such as sucrose and CO_2_ (Arikawa et al., 2017; Tanaka et al., 2021). The strain *R. eutropha* Re2058/pCB113 was engineered with an heterologous PHA synthase (phaC2 from *Rhodococcus aethivorans*) and an enoyl-coA hydratase (phaJ1 from *Pseudomonas aeruginosa*) to accumulate P(HB-*co*-HHx) when fed with raw materials containing fatty acids (Budde, et al., 2011a; Riedel et al., 2012; Saad et al., 2021; Gutschmann et al., 2022b), whereas it accumulates only PHB when fed with sugars (Santolin et al., 2021). The utilization of oily substrates by the strain is realized via the natural secretion of lipases that mediate the hydrolysis of the triacylglycerols forming natural emulsions that may also be stabilized by extracellular polysaccharides (Gutschmann et al., 2021). The great interest reported on P(HB-*co*-HHx) relates with the possibility of tailoring the physiochemical properties of this bioplastic targeting specific applications by adjusting the HHx molar fraction of the copolymer (Selli et al., 2022).

In the current study we report a very simple batch strategy that enabled to precisely control the HHx molar content in tailor-made P(HB-*co*-HHx) copolymers employing varying mixtures of fructose and canola oil with a fixed final carbon content for better comparison. An upscale of the method from the mL- to the L-scale starting in deep-well-plates and moving on to shake flasks and finally to lab-scale bioreactors proves the robustness of our approach.

## 2 Materials and methods

### 2.1 Bacterial strain

All experiments were conducted with the engineered *R. eutropha* strain Re2058/pCB113 that produces the copolymer P(HB-*co*-HHx) when grown on oleaginous feedstocks (Budde, et al., 2011b). The strain was stored in 20% (v v^-1^) glycerol at −80°C.

### 2.2 Seed train

Tryptic soy broth (TSB) media, agar plates and mineral salt media (MSM) compositions have been described previously (Gutschmann et al., 2019). *Ralstonia eutropha* Re2058/pCB113 was streaked from a cryoculture on a TSB agar plate and incubated for 3–4 days at 30°C. A single colony from the plate was used to inoculate 10 mL TSB using a 125-mL Ultra Yield Flask (Thomson Instrument Company, United States), equipped with an AirOtop membrane (Thomson Instrument Company, United States). TSB was always supplemented with 10 μg mL^−1^ gentamycin sulfate and 200 μg mL^−1^ kanamycin sulfate. The preculture was incubated at 30°C and 200 rpm shaking speed for approximately 17 h or until an OD_600_ of 5 was reached. The main cultures in MSM were inoculated to an initial OD_600_ of 0.05. Fructose or canola oil (Edeka Zentrale AG and Co. KG, Germany) were used as carbon sources and urea was used as the sole nitrogen source in the MSM. The explicit amounts are described in the text. All chemicals were purchased from Carl Roth GmbH and Co. KG (Germany) unless stated otherwise.

### 2.3 Calculation of C/N ratio

Specific carbon and nitrogen concentrations (g L^−1^) and carbon-to-nitrogen ratios [C/N (g g^−1^)] were used for all experiments. See Supplementary Material for calculations.

### 2.4 Deep-well-plate cultivations

24-deep-well-plates with square shape wells and a maximum volume of 11 mL (Duetz-MTPS, Adolf Kühner AG, Switzerland) were used in this study. To ensure identical cultivation conditions for the deep-well-plate replicates, 50 mL of each media with each chosen fructose to canola oil ratio was prepared and inoculated from TSB overnight cultures (see above), and then 3 mL of culture was transferred into each of the wells. To obtain a defined and comparable canola oil concentration in the different wells, the medium was pre-emulsified with gum arabic (GA) before sterilization using an adapted method from Budde et al. (2011a): each medium was prepared by mixing the phosphate buffer, water and K_2_SO_4_ with the desired amount of canola oil and adding GA to a final concentration 0.3% (w v^−1^). The mixture was homogenized with an Ultra-Turrax T25 (IKA-Werke GmbH and Co. KG, Germany) for 1 min at 8,000 rpm. After emulsifying the oil, the media was autoclaved, and the remaining media components were added from sterile stocks. GA was chosen as the emulsifier as it has been shown not to support growth of *R. eutropha* (see Supplementary Figure S1). Plates were incubated for 72 h at 30°C and 225 rpm in an orbital shaker with 50 mm amplitude. Culture volume and incubation conditions were chosen according to manufacturer’s instructions to ensure sufficient oxygen supply. Biological triplicates were performed for each condition.

#### 2.4.1 Evaluation of suitable C/N ratio

In the first series of experiments, growth at four different carbon concentrations: 0.5%, 1%, 1.5%, and 2% (w v^−1^) of fructose and total carbon equivalent concentrations of canola oil: 0.25%, 0.5%, 0.75%, and 1% (w v^−1^) was evaluated with a fixed amount of 0.744 g L^−1^ urea as nitrogen source, resulting in C/N ratios of about 5, 11, 17, and 22 (g g^−1^). For increased C/N ratios of about 22, 45, 68, and 90 (g g^−1^), the same carbon concentrations were tested with 0.186 g L^−1^ urea.

#### 2.4.2 Evaluation of different fructose to canola oil mixtures

A regular distribution of different ratios of canola oil to fructose as well as each sole carbon source were tested to determine their effect on cell growth and PHA accumulation and composition. Seven different mixtures of fructose and canola oil, namely, 1:0, 5:1, 2:1, 1:1, 0.5:1, 0.2:1, and 0:1 [carbon ratio fructose to canola oil (g g^−1^)], all yielding a final carbon content of 5 g L^−1^ were used in combination with 0.46 g L^−1^ urea to reach the selected C/N ratio of 22 (g g^−1^).

### 2.5 Shake flask cultivations

Four different mixtures of fructose and canola oil were selected from the deep-well-plate cultivations and upscaled to 100 mL cultures, applying the exact same cultivation strategy at this scale. Cultivations were performed in 500-mL DURAN baffled flasks (DWK Life Science GmbH, Germany) sealed with AirOtop membranes to ensure sufficient oxygen supply. These cultivations, performed in biological triplicates, were incubated at 30°C and 200 rpm in an orbital shaker (50 mm amplitude) for 72 h and sampling was performed every 24 h. The cultivations were then repeated doubling the amount of carbon content available to 10 g L^−1^ but maintaining the C/N ratio by also doubling the urea concentration to 0.92 g L^−1^.

### 2.6 Bioreactor cultivations

P(HB-*co*-HHx) production with fructose and canola oil mixtures was upscaled to 1-L bioreactors using six Multifors2 parallel benchtop bioreactors with two six-blade Rushton impellers (Infors AG, Switzerland). The cultivation temperature was kept constant at 30°C and the pH was maintained at 6.8 ± 0.1 using 1 M H_3_PO_4_ and 2 M NaOH for pH control. The initial stirring speed was set to 200 rpm, whereas the initial flow rate was set to 0.05 vvm. Via an automatized cascade, aeration was increased up to 0.5 vvm and later stirring was increased up to 1,500 rpm in order to prevent DO values from dropping below 40%. Foam was mechanically broken as described previously (Riedel et al., 2012). Six different mixtures of fructose and canola oil, all yielding a final carbon content of 10 g L^−1^ and a C/N ratio of 22 (g g^−1^) were used to produce sufficient amounts of P(HB-*co*-HHx) copolymers with varying HHx monomer content for polymer characterization.

### 2.7 Analytical methods

For quantification, the entire 3 mL culture was taken from the deep-well-plates at the end of the cultivations, while for cultivations in shake flasks and bioreactors, 5 mL samples were taken at each sampling point. For cell dry weight (CDW) determination the samples were collected in pre-weighed 15-mL tubes and centrifuged for 15 min and 4°C at 8,000 × *g*. The pellets were washed with 3.5 mL cold deionized (DI) water and 1.5 mL cold hexane to remove residual oil and then resuspended again in 2 mL DI water, frozen at −80°C and dried for 48 h by lyophilization (Gamma 1–20, Martin Christ Gefriertrocknungsanlagen GmbH, Germany).

The PHA content and composition of the dried cells were determined using a methanolysis protocol and gas chromatography as previously described (Bartels et al., 2020). Residual cell dry weight (RCDW) was defined as CDW minus PHA content in g L^−1^.

During bioreactor cultivations, fructose and NH_3_ concentrations were measured from the supernatant of centrifuged samples. For fructose measurement, 750 µL of supernatant was washed twice by mixing with 750 µL of cold hexane in a 2-mL Eppendorf tube and shaking for 15 min in an overhead shaker (Rotator Drive STR4, StuartScientific, Cole-Parmer, Germany). Centrifugation was performed at 8,000 × *g* for 2 min and the bottom phase was collected. The washed supernatant was then filtered through an 0.2 µm PES syringe filter and fructose concentration determined via HPLC-RID. Chromatography was performed with 20 µL injection volume at 80°C for 62 min on an Agilent Hi-Plex Ca column. The eluent was DI H_2_O with an 0.6 mL min^−1^ flux. Unfiltered and unwashed supernatant was measured using the Cedex Bio HT Analyzer (Cedex Bio HT Analyzer, Roche Diagnostics International AG, Switzerland) to determine NH_3_ consumption.

### 2.8 Determination of molecular weight characteristics of the produced PHA

Molecular weight distribution of the PHA polymers was determined by size exclusion chromatography (SEC) from CDW samples as described previously (Thiele et al., 2021).

## 3 Results

### 3.1 Determination of a suitable C/N ratio in deep-well-plate cultivations

**FIGURE 1:**
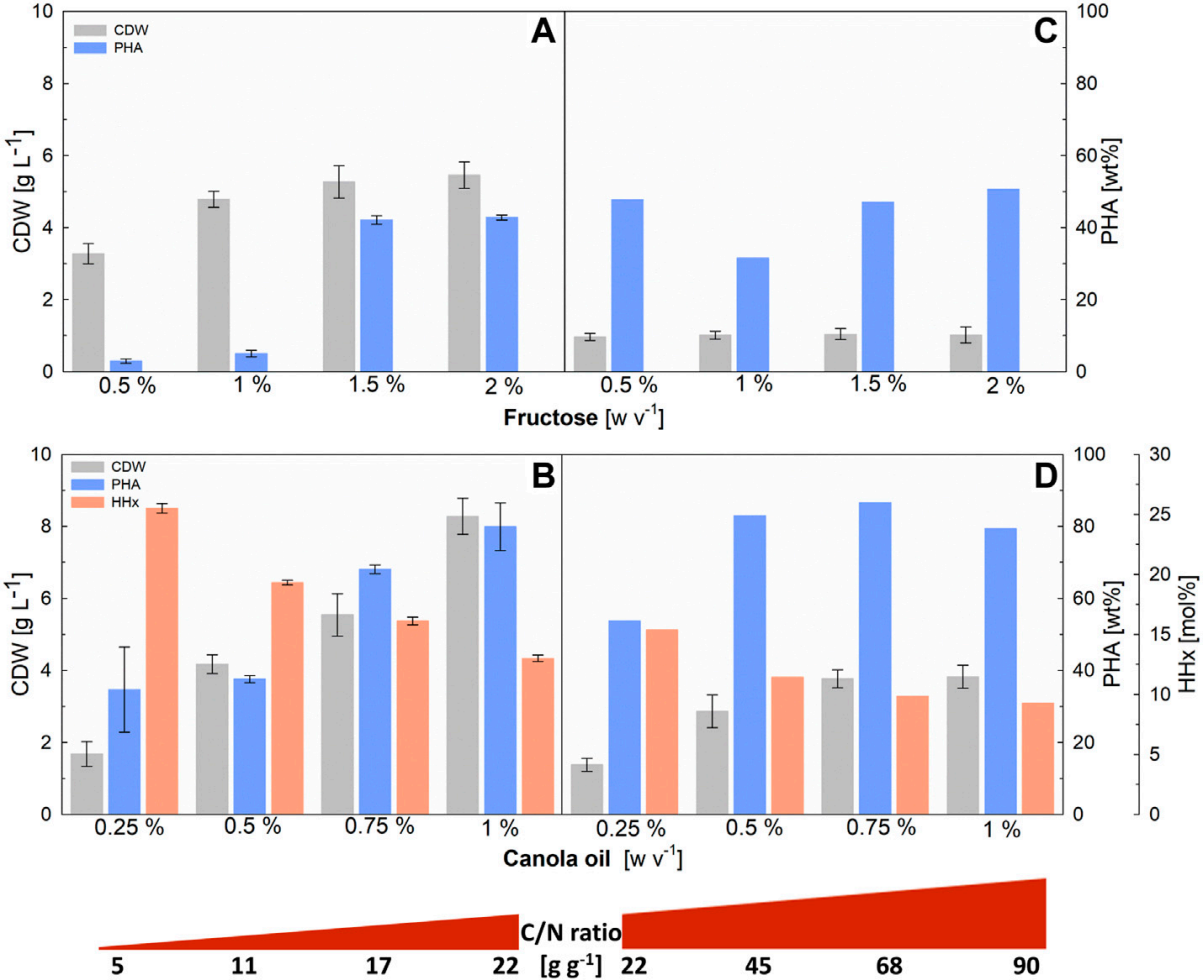
Evaluation of C/N ratios in 3-mL deep-well-plates for *R. eutropha* Re2058/pCB113 cultivations using fructose or canola oil as carbon source and urea as nitrogen source. Cell dry weight (CDW; g L^−1^), PHA content of CDW (PHA; wt%) and HHx content of PHA (HHx; mol%) achieved after 72 h of cultivation with different C/N ratios (g g^−1^) are shown. Left graphs show the results of low C/N ratios achieved with 0.744 g L^−1^ urea and different (w v^−1^) concentrations of fructose **(A)** and total-carbon-equivalent canola oil concentrations **(B)**. Right graphs show the results of high C/N ratios obtained with the same fructose **(C)** and canola oil **(D)** concentrations and 0.186 g L^−1^ urea. CDW error bars indicate standard deviation from biological triplicates. PHA and HHx error bars represent standard deviation from duplicate measurements of pulled samples [in **(C,D)** the scarce amount of sample was only sufficient for a single measurement].

The choice of a suitable C/N ratio is crucial for matching the monomer composition of P(HB-*co*-HHx) to the substrate mixture supplied as, with excess carbon sources, only the preferred substrate will be used so that the effects of different mixing ratios will be negligible. Different C/N ratios, in the range of 5–90 (g g^−1^) were investigated in deep-well-plates with a working volume of 3 mL and the results are shown in Figure 1.

Increasing final biomass and PHA content values were observed with increasing C/N ratios from 5 to 22 (Figures 1A, B). For fructose, no increase in these values was observed when the C/N ratio was further increased above 22 (Figure 1C), indicating that the added fructose was not consumed. In the case of canola oil (Figure 1D), a stagnation of the achieved biomass was only observed above a C/N ratio of 68 (g g^−1^). PHA values showed, that cells growing on fructose accumulated only up to about 50 wt% of PHA, while cells growing on canola oil were able to accumulate up to 80 wt% of PHA, which explains why, with canola oil, more carbon source was consumed and final biomass values increased with increasing C/N ratios above 22 (g g^−1^). As expected, the final CDW values were about four times higher in the first series of experiments (Figures 1A, B), where the added urea concentration was four times higher than in the second series of experiments (Figures 1C, D), with the same C/N ratio.

For the following experiments with fructose and canola oil mixtures, a C/N ratio of 22 (g g^−1^) was chosen, as both carbon sources were to be completely consumed in order to determine the effects of different mixture ratios on the HHx content.

### 3.2 Evaluation of the impact of different mixtures of fructose and canola oil on the P(HB-*co*-HHx) composition in deep-well-plate cultivations

**FIGURE 2:**
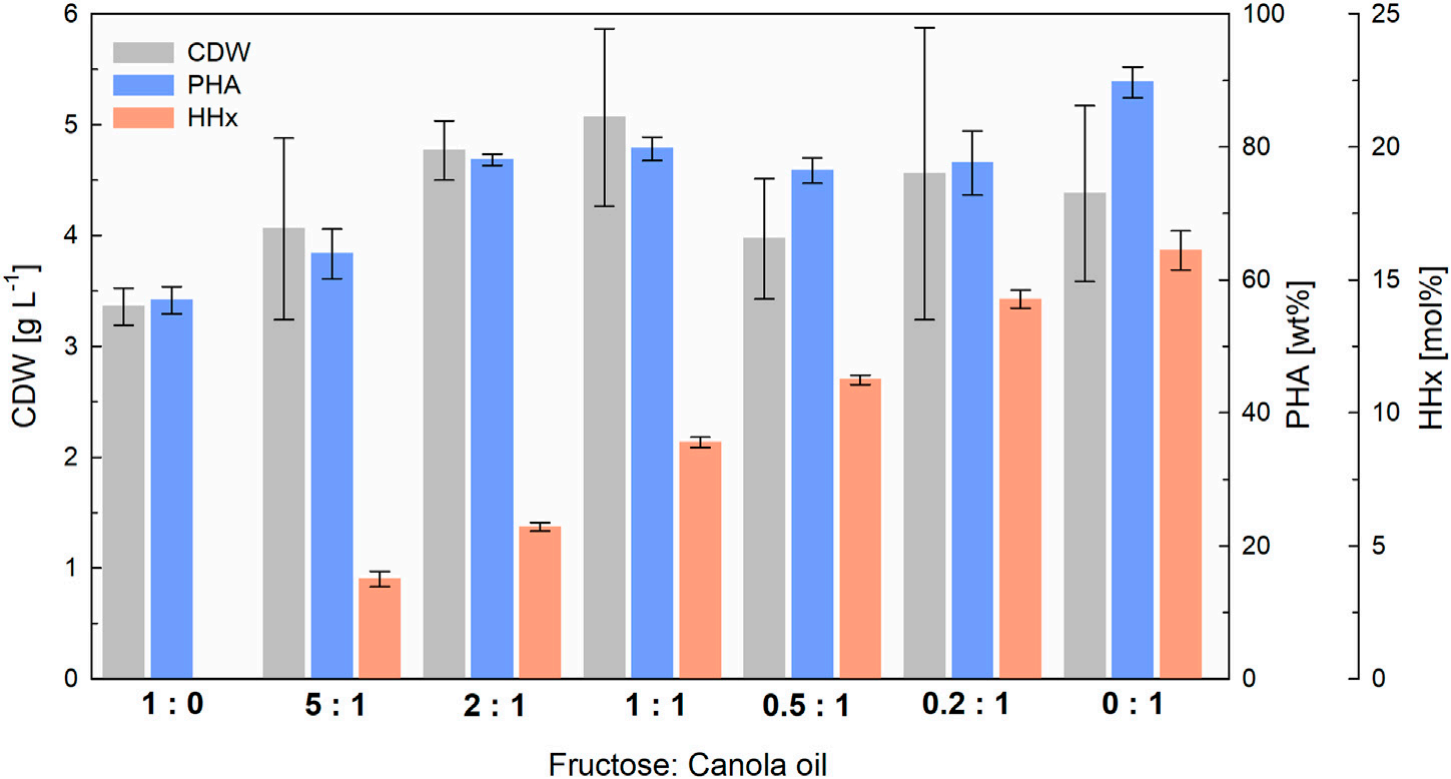
Impact of different fructose to canola oil mixture ratios on the composition of P(HB-*co*-HHx) in 3-mL deep-well-plate cultivations with *R. eutropha* Re2058/pCB113 using urea as nitrogen source. Cell dry weight (CDW; g L^−1^), PHA content of CDW (wt%) and HHx content of PHA (mol%) achieved after 72 h with different mixtures with a final carbon content of 5 g L^−1^ and a C/N ratio of 22 g g^−1^ are displayed. The carbon ratio (g g^−1^) of fructose to canola oil is indicated for each mixture. CDW error bars indicate standard deviation from biological triplicates. PHA and HHx error bars represent standard deviation from duplicate measurements of pulled samples.

Seven fructose and canola oil mixtures, all with a total carbon content of 5 g L^−1^, were used to test the effect of varying substrate ratios on the molar compositions of P(HB-*co*-HHx) in 24-deep-well-plates with a working volume of 3-mL (see Figure 2). Depending on the amount of canola oil, the HHx content increased linearly from 0 mol% when no oleaginous feedstock was available to 16 mol% with canola oil as the sole carbon source (see Supplementary Figure S2 for linear correlation). Comparable final biomass values over 4 g L^-1^ with 65–90 wt% PHA were observed with all mixtures except when fructose was supplied as the sole carbon source. With a maximum accumulation of 57 wt% PHA, the final biomass values of pure fructose cultures showed the lowest CDW accumulation.

### 3.3 Upscaling and optimization of mixed substrate cultivations to shake flask scale

**FIGURE 3:**
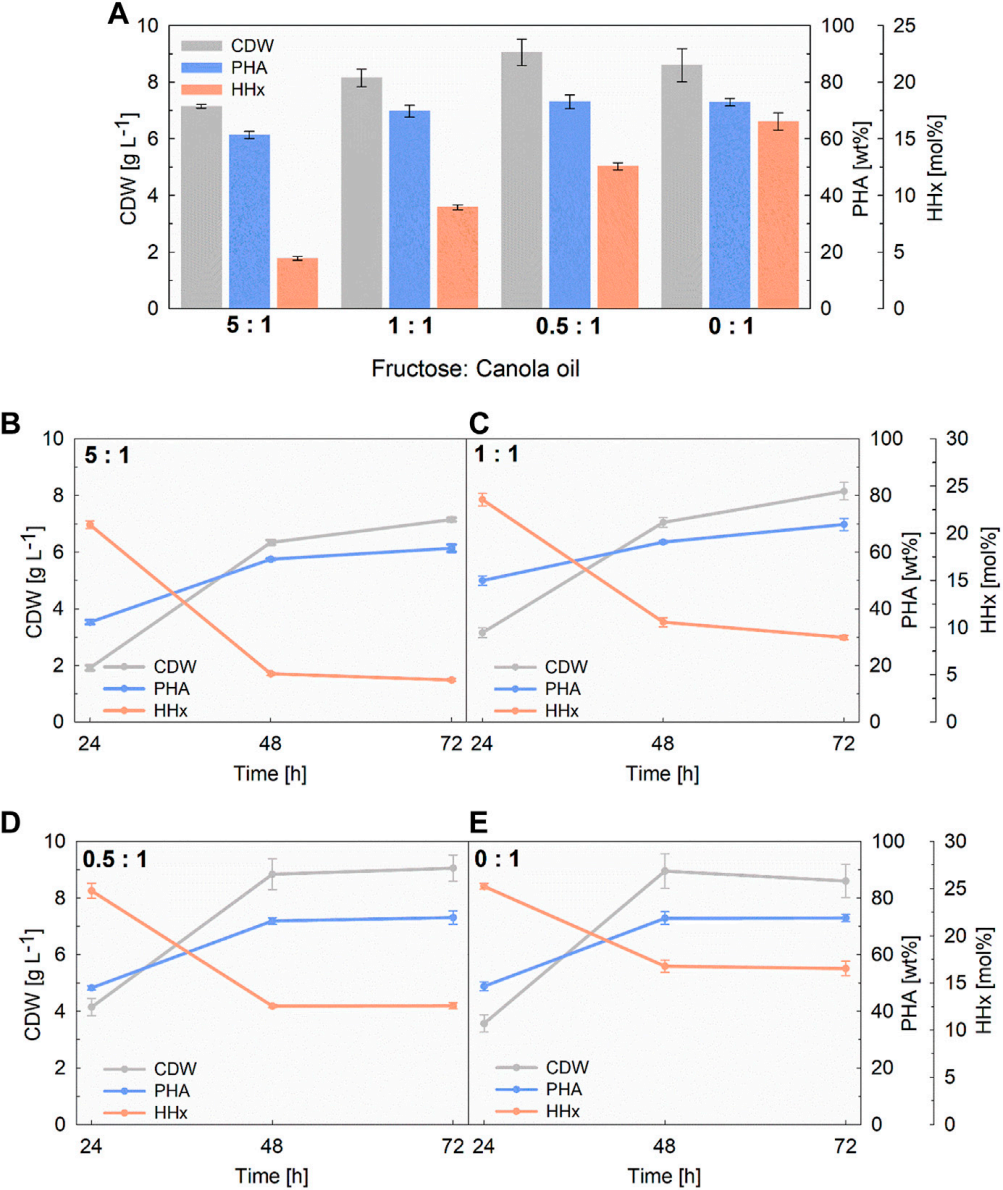
100-mL shake flask cultivations with *R. eutropha* Re2058/pCB113 using fructose and canola oil mixtures as carbon source and urea as nitrogen source. Final yields of cell dry weight (CDW; g L^−1^), PHA content of CDW (PHA; wt%) and HHx content of PHA (HHx; mol%) after 72 h are shown in **(A)** as well as values every 24 h for each cultivation with 5: 1 **(B)**, 1: 1 **(C)**, 0.5: 1 **(D)**, and 0: 1 **(E)** fructose to canola oil ratio. All cultivations had a final carbon content of 10 g L^−1^ with a C/N ratio of 22 g g^−1^. The carbon ratio of fructose to canola oil (g g^−1^) is indicated for each mixture. Error bars indicate standard deviation from biological triplicates.

Four of the previously tested mixtures, namely, 5:1, 1:1, 0.5:1, and 0:1 [carbon ratio fructose to canola oil (g g^−1^)], were scaled up, following the same cultivation strategy (C/N ratio and total carbon content), to 100 mL working volume in shake flask cultivations. When utilizing the same final carbon content as in deep-well-plates cultivations (5 g L^−1^), comparable biomass values were obtained (see Supplementary Figure S3). Nevertheless, in the three mixtures with the higher canola oil contents, a decrease in the PHA content between 48–72 h was observed showing also an increased HHx content at the end of the cultivation in comparison with the previous deep-well-plate experiments. To avoid premature degradation of the PHA granules and to achieve higher final yields, it was decided to double the used carbon content to 10 g L^−1^ while maintaining the C/N ratio of 22 (g g^−1^) (see Figure 3). With this approach, comparable results were achieved in terms of PHA content and composition as with the deep-well-plate cultivations, while the final biomass yield was approximately doubled (see Table 1).

**TABLE 1:** Comparison of biomass, PHA content and composition obtained among all studied scales with *R. eutropha* Re2058/pCB113 using mixtures of fructose and canola oil as carbon source and urea as nitrogen source with an applied C/N ratio of 22 g g-1. For deep-well-plate cultivations, CDW measurements represent means from triplicate cultivations and PHA and HHx measurements represent means from duplicate measurements of pulled samples. For shake flask cultivations, measurements represent means of triplicate cultivations. ± are indicating standard deviation.

Fructose: Canola oil [g g^−1^]	Scale	Total carbon [g L^−1^]	CDW [g L^−1^]	PHA [wt%]	HHx [mol%]
**1: 0**	3-mL deep-well-plate	5	3.4 ± 0.2	56.9 ± 2.0	0.0
**10: 1**	1-L bioreactor	10	9.7	66.5	2.3
**5: 1**	3-mL deep-well-plate	5	4.1 ± 0.8	63.9 ± 3.7	3.7 ± 0.3
	100-mL shake flask	5	3.9 ± 0.3	57.3 ± 4.7	4.1 ± 0.5
	100-mL shake flask	10	7.1 ± 0.1	61.3 ± 1.3	4.4 ± 0.2
	1-L bioreactor	10	9.0	58.6	4.3
**2: 1**	3-mL deep-well-plate	5	4.8 ± 0.3	78.1 ± 0.9	5.7 ± 0.2
	1-L bioreactor	10	10.5	60.4	6.5
**1: 1**	3-mL deep-well-plate	5	5.1 ± 0.8	79.7 ± 1.8	8.9 ± 0.2
	100-mL shake flask	5	4.6 ± 0.4	63.6 ± 2.5	9.2 ± 0.2
	100-mL shake flask	10	8.1 ± 0.3	69.7 ± 2.1	8.9 ± 0.2
	1-L bioreactor	10	10.9	70.4	7.5
**0.5: 1**	3-mL deep-well-plate	5	4.0 ± 0.5	76.4 ± 1.9	11.2 ± 0.2
	100-mL shake flask	5	4.7 ± 0.2	65.3 ± 3.0	14.6 ± 0.2
	100-mL shake flask	10	9.0 ± 0.5	73.1 ± 2.4	12.6 ± 0.3
	1-L bioreactor	10	12.3	75.7	11.4
**0.2: 1**	3-mL deep-well-plate	5	4.6 ± 1.3	77.6 ± 4.8	14.3 ± 0.7
**0: 1**	3-mL deep-well-plate	5	4.4 ± 0.8	89.7 ± 2.3	16.1 ± 0.7
	100-mL shake flask	5	4.0 ± 0.3	66.5 ± 2.6	21.9 ± 1.4
	100-mL shake flask	10	8.6 ± 0.6	72.9 ± 1.3	16.5 ± 0.8
	1-L bioreactor	10	12.9	88.0	14.3

### 3.4 Upscaling the production of molar-specific P(HB-*co*-HHx) to 1-L bioreactor cultivations

**FIGURE 4:**
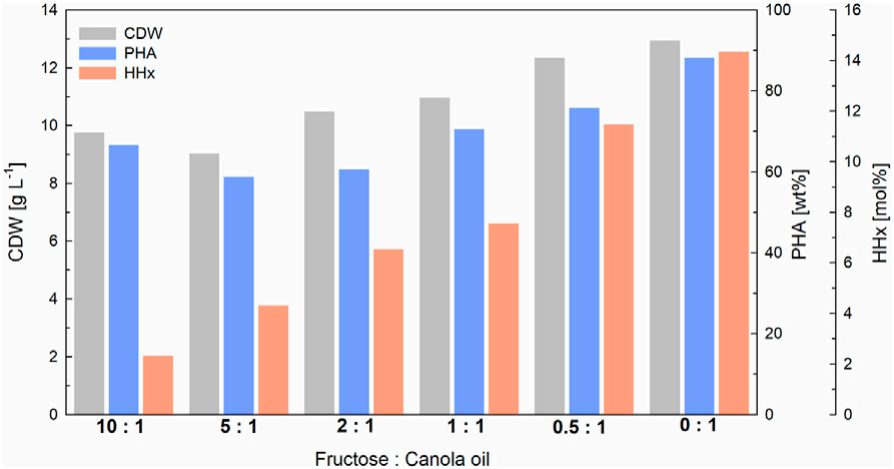
Final yields of 1-L bioreactor cultivations for tailor-made P(HB-*co*-HHx) production with *R. eutropha* Re2058/pCB113 using fructose and canola oil mixtures as carbon source and urea as nitrogen source. Cell dry weight (CDW; g L^−1^), PHA content of CDW (PHA; wt%) and HHx content of PHA (HHx; mol%) after 72 h are shown for each mixture with a final carbon content of 10 g L^−1^ and a C/N ratio of 22 g g^−1^. The carbon ratio of fructose to canola oil (g g^−1^) is indicated for each mixture.

**FIGURE 5:**
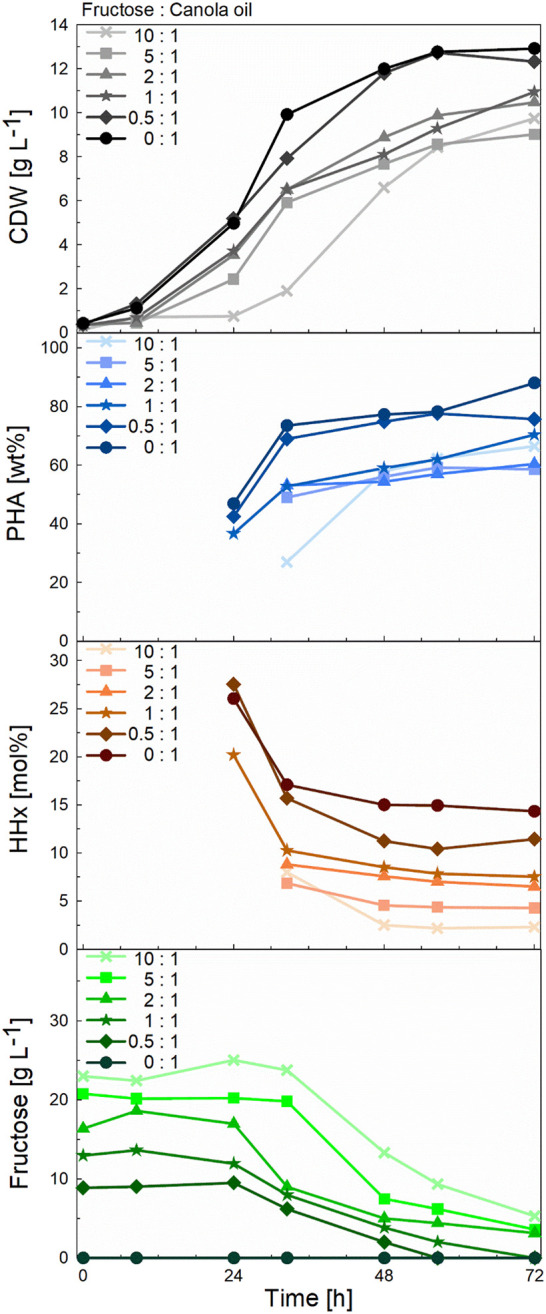
Comparison of CDW, PHA, and HHx accumulation- and fructose consumption curves during 1-L bioreactor cultivations of *R. eutropha* Re2058/pCB113 for tailor-made P(HB-*co*-HHx) production using mixtures with decreasing fructose to canola oil ratios as carbon source and urea as nitrogen source. Cell dry weight (CDW; g L^−1^), PHA content of CDW (PHA; wt%), HHx content of PHA (HHx; mol%) and fructose concentration (Fructose; g L^−1^) values are shown over the course of the cultivation for each mixture with a final carbon content of 10 g L^−1^ and a C/N ratio of 22 g g^−1^. The carbon ratio of fructose to canola oil (g g^−1^) is indicated for each mixture.

To demonstrate the scalability of our approach, 1-L bioreactor cultivations were carried out transferring the cultivation strategy from shake-flask cultivations (constant C/N ratio and total carbon content). Again, a linear correlation was found between the amount of canola oil and fructose used and the HHx content obtained (see Supplementary Figure S2). With final biomass values between 9–13 g L^−1^ and PHA contents between 60–88 wt%, the final yields were slightly better than in the previous shake flask cultures (Figure 4). The data showed that fructose was not consumed at least during the first 24 h of cultivation, suggesting that canola oil was consumed first, and the cells later switched to fructose as the non-preferred carbon source (Figure 5). In the three cultures with the highest fructose ratios, residual fructose concentrations were measured between 3–5 g L^-1^, indicating that the cells would have reached slightly higher PHA contents with slightly lower HHx molar contents if the cultivations had been operated for a longer period. In these three cultures nitrogen depletion also set in later than in cultures with higher canola oil ratios (see Supplementary Figure S4).

### 3.5 Comparison between deep-well-plate-, shake flask- and bioreactor-scale

When comparing the final CDW values at all scales investigated, a slight increase in biomass yields was observed when moving from deep-well-plates to shake flasks and from shake flasks to bioreactor cultivations (see Table 1). Accordingly, when twice the total amount of carbon was added in the second shake flask run, the final CDWs approximately doubled. Within each scale, biomass values were comparable throughout all mixtures, whereby the values were slightly higher with higher canola oil contents. PHA values were comparable at all scales, with a general increase in polymer accumulation (from 60%–90%) measured with increasing canola oil contents. A linear correlation between the canola oil content in each mixture and the final HHx molar content was observed at all scales (see Supplementary Figure S2) showing that it was possible to tailor P(HB-*co*-HHx) by only applying different fructose and canola oil mixtures. When higher polymer yields were obtained (shake flask with 10 g L^−1^ CC vs. shake flask with 5 g L^−1^ CC and bioreactor vs. shake flask with 10 g L^−1^ CC), slightly lower molar HHx contents were observed.

### 3.6 Characterization of P(HB-*co*-HHx) copolymers

Molecular weight characteristics of the produced copolymers after 72 h of cultivation in shake flask and bioreactor scale were determined by size exclusion chromatography. A decrease of the molecular weight with increasing scale was noticeable, whereas no marked decrease in M_w_ was observed with increasing HHx contents (Table 2). Only the shake flask cultivations containing 5 g L^−1^ carbon as substrate showed a slight decrease in the M_w_ of about 10% with increasing HHx molar fraction. When the final carbon content was doubled from 5–10 g L^−1^ in shake flask cultivations, a clear decrease in the final M_w_, up 35% was observed. In bioreactor cultivations, where higher PHA contents per CDW were reached compared to shake flask cultivations, the lowest molecular weights around 3.5 × 10^5^ Da were obtained. The polydispersity index Đ, around 2.5, was comparable along the scales. During bioreactor cultivations no clear decrease in the M_w_ was observed over time (see Supplementary Table S1).

**TABLE 2:** Molecular weight characterization of samples after 72 h of 100-mL shake flask and 1-L bioreactor cultivations with R. eutropha Re2058/pCB113 using mixtures of fructose and canola oil and urea as nitrogen source with an applied C/N ratio of 22 g g-1. Mw = weight-average molecular weight, Mn = number-average molecular weight, Đ = polydispersity index. Measurements represent means from duplicate measurements. ± are indicating minimum and maximum values.

Fructose: Canola oil [g g^−1^]	Scale	Total carbon [g L^−1^]	M_w_ × 10^5^ [Da]	M_n_ × 10^5^ [Da]	Đ [-]
**10: 1**	1-L bioreactor	10	3.51	1.42	2.47
**5: 1**	100-mL shake flask	5	6.2 ± 0.1	2.5 ± 0.3	2.5 ± 0.2
	100-mL shake flask	10	4.0 ± 0.3	1.6 ± 0.2	2.5 ± 0.2
	1-L bioreactor	10	4.0 ± 0.1	1.6 ± 0.1	2.4 ± 0.0
**2: 1**	1-L bioreactor	10	3.2 ± 0.0	1.3 ± 0.0	2.4 ± 0.0
**1: 1**	100-mL shake flask	5	6.1 ± 0.1	2.8 ± 0.1	2.2 ± 0.0
	100-mL shake flask	10	4.8 ± 0.7	1.8 ± 0.1	2.6 ± 0.3
	1-L bioreactor	10	3.5 ± 0.0	1.5 ± 0.0	2.3 ± 0.0
**0.5: 1**	100-mL shake flask	5	5.8 ± 0.1	2.7 ± 0.1	2.2 ± 0.0
	100-mL shake flask	10	4.8 ± 0.3	2.2 ± 0.3	2.2 ± 0.2
	1-L bioreactor	10	3.6 ± 0.1	1.7 ± 0.1	2.2 ± 0.1
**0: 1**	100-mL shake flask	5	5.6 ± 0.1	2.5 ± 0.3	2.5 ± 0.2
	100-mL shake flask	10	4.2 ± 0.8	1.6 ± 0.9	2.1 ± 0.1
	1-L bioreactor	10	3.4 ± 0.1	1.5 ± 0.0	2.3 ± 0.0

## 4 Discussion

We have developed a simple and robust batch strategy to control the molar HHx content in P(HB-*co*-HHx) from the 3-mL deep-well-plate to 1-L bioreactor scale. Since *R. eutropha* Re2058/pCB113 produces the copolymer only when grown on oleaginous feedstocks but not when using sugars (Budde et al., 2011b), mixtures of fructose and canola oil were chosen to tune the monomer composition of P(HB-*co*-HHx). While most published studies to date vary the concentration of one substrate while using a fixed concentration of the respective sugar or oleaginous feedstock (Murugan et al., 2016; Murugan et al., 2017; Purama et al., 2018), we orientated our studies on consistently using the same total carbon content and C/N ratio for all mixtures, achieving comparable CDWs and PHA contents with all mixtures that were reproducible along all scales (7–13 g L^−1^ CDW and 60–88 wt% PHA respectively). The strategy succeeded in tuning HHx monomer contents from 2–17 mol%, showing a linear correlation, validated at all scales, with the canola oil content in each mixture.

It has been shown that carbon sources containing a higher abundance of MCFAs (medium-chain fatty acids, 6–12 carbons) lead to a higher incorporation of HHx precursors than using plant oils holding LCFAs (long-chain fatty acids, 13–21 carbons) like canola oil as used in this study (Mifune et al., 2008; Budde et al., 2011a). Per fatty acid, only one molecule of 3HHx-CoA can be formed, thus shorter fatty acids lead to a lower ratio of 3HB-CoA to 3HHx-CoA as fewer acetyl-CoA molecules are released from β-oxidation (Riedel et al., 2014). Date seed oil with 19.1% C12:0 (lauric acid) or crude palm kernel oil (CPKO) containing 3% C8:0, 3% C10:0%, and 48% C12:0 produced P(HB-*co*-HHx) with 39 and 44 mol% HHx, respectively (Murugan et al., 2016; Purama et al., 2018). Using CPKO in combination with oil palm tree trunk sap in shake flask cultivations, Murugan et al. obtained from 31 up to 68 wt% of PHA at 4.2–7.1 g L^−1^ CDW, and comparatively higher HHx molar ratios from 14–27 mol% (Murugan et al., 2016). In a follow up study, three substrate mixtures of palm olein and fructose were chosen, to obtain P(HB-*co*-HHx) with lowered HHx contents from 4–15 mol% in bioreactor cultivations (Murugan et al., 2017). Here, the effect of increasing the sugar to oil ratio to effectively lower the HHx fraction was proved as palm tree trunk sap, containing only 17% fructose of the total sugars with a large fraction of glucose, was replaced by pure fructose. *Ralstonia eutropha* is only able to metabolize fructose and no glucose (Sichwart et al., 2011). Further, date molasses, containing over 50% fructose, and date seed oil mixtures were also used in bioreactor cultivations reaching varying CDW concentrations from 1.7–6.9 g L^−1^ CDW, up to 49 wt% of PHA and broader HHx molar ratios from 2–28 mol% (Purama et al., 2018).

All abovementioned studies, even if they achieved lower biomass yields due to the use of less urea as nitrogen source (about half of this study) and less comparability along the tested mixtures, are based on the same principle and prove that it is plausible to extend our strategy to other feedstocks. Another different approach to control the HHx fraction of P(HB-*co*-HHx) on a molecular level was presented by controlling the expression of the *phaJ* gene of *R. eutropha*, involved in the generation of HHx precursors, showing that copolymers with HHx molar contents ranging from 2.8–10.7 mol% could be obtained (Miyahara et al., 2021). Arikawa et al. recently reported the tailored production of P(HB-*co*-HHx) with HHx contents up to 36 mol% by the deletion of the *ß*-ketothiolase gene together with the overexpression of the (R)-specific enoyl-Coa hydratase and PhaC synthase (Arikawa and Sato, 2022).

As in the studies mentioned above, an increase in the molar HHx content was observed in mixtures with increasing oleaginous substrate concentrations. Furthermore, in accordance with the literature (Budde et al., 2011a; Riedel et al., 2015), a decrease in the HHx content was measured over the course of the cultivation. Whether growing on a pure oleaginous feedstock or in combination with fructose, during the growth phase less HB precursors will be formed in comparison to the production phase as acetyl-coA flows into the TCA cycle and less HB precursors are formed. In this context, it is reported that high intracellular CoA concentrations inhibit PhaA, leading to a slower rate of HB-CoA synthesis (Oeding and Schlegel, 1973). When PHA storage is triggered by nutrient limitation or stress conditions, more HB precursors will be incorporated into the polymer, gradually levelling off the relative concentration of HHx monomers. Additionally, oil is preferred over sugars which contributes to the higher contents of HHx at the beginning of the cultivation when this substrate is being consumed. Monitoring of the fructose concentration in the medium showed that fructose was not consumed at least in the first 24 h of cultivation, which was also observed in other studies (Murugan et al., 2017). The fact that no dissolved oxygen peak (or dropping of the stirring cascade) was observed around the timepoint when the strain started to consume fructose (data not shown) as the second preferred carbon source suggests a smooth transition from one substrate to the other with the strain presumably being able to assimilate both canola oil and fructose simultaneously.

In general, a higher PHA content of up to 88 wt% was obtained when the carbon source was of oleaginous origin, whereas only about 60 wt% PHA could be obtained with fructose alone as substrate. This is due to the fact that the utilization of fructose in this strain is less efficient than the utilization of oleaginous feedstocks. After the conversion of fructose to two pyruvate molecules via the Entner-Doudoroff pathway, one molecule of CO_2_ is released for the conversion to each acetyl-CoA by the pyruvate-dehydrogenase while no carbon in the form of CO_2_ is lost in the ß-oxidation of oils. In addition, the strain Re2058/pCB113 was engineered to utilize plant oils efficiently, boosting the synthesis of HHx monomers (Budde et al., 2011b).

When higher polymer yields were obtained (shake flasks with 5 g L^−1^ carbon content vs. shake flasks with 10 g L^−1^ carbon content and shake flasks with 10 g L^−1^ carbon content vs. bioreactor cultivations), slightly lower molar HHx contents were observed. This could be due to the degradation of the HB-rich polymer ends after 48 h in the first case, which was observed when only half of carbon was applied, presumably due to depletion of the carbon sources. In the second case, a faster growth in the bioreactor in comparison to the shake flasks supported by a better overall physiological state (pH- and O_2_-control) may have enabled the cells to further consume the fructose present in the media, leading to more HB-monomers being incorporated to the polymer chain, thus decreasing the final HHx content.

During cultivations in the bioreactor, CDW values were obtained that were higher than the theoretical yield, which can be explained by evaporation during sterilization of the media and thereby a concentration of the carbon sources, as well as by a slight misestimation of the carbon content of canola oil (Table 1).

In this study, no significant change in the molecular weight with different HHx fractions was observed when the carbon source concentration was 10 g L^−1^. In the shake flask cultivation, where a lower substrate concentration of 5 g L^−1^ was used (Supplementary Figure S3), a decrease of the molecular weight of the copolymer with increasing HHx fraction was observed, contradicting the results obtained by Murugan et al. (2017) who obtained an increasing M_w_. Purama et al. (2018) reported an overall decreasing trend of molecular weights between 8.3 and 5.8 × 10^5^ Da with increasing HHx fractions between 5 and 28 mol%. Additionally, they observed a narrower polydispersity index of 1.7 compared to our study, which was around 2.17–2.61. Generally, the bulkier HHx is assumed to reduce the synthase turnover rate leading to lower molecular weights (Murugan et al., 2017). Moreover, when the substrate concentration was doubled, the glycerol concentration from oil cleavage by secreted lipases of *R. eutropha* also increased. Glycerol reportedly acts as a chain terminator and could thus be the cause of the lower molecular weight observed during these cultivations (Ashby et al., 2012).

## 5 Conclusion

The simple and robust approach presented in this study using mixtures of fructose and canola oil can be used to produce P(HB-*co*-HHx) with precisely controlled compositions. The strategy, which allows the HHx content to be adjusted between 2–17 mol%, proved to be scalable from the mL-scale in deep-well-plates to the L-scale in bioreactors. At all scales, high PHA contents of over 60 wt% were obtained with comparable molecular weight properties. However, to increase the overall yields of tailor-made P(HB-*co*-HHx), a fed-batch process needs to be developed for the different substrate mixtures.

## Data Availability

The raw data supporting the conclusion of this article will be made available by the authors, without undue reservation.
