# Supplementary material for: Tailoring the HHx monomer content of P(HB-co-HHx) by flexible substrate compositions: scale-up from deep-well-plates to laboratory bioreactor cultivations
Source: Front Bioeng Biotechnol. 2023 May 2;11:1081072. doi: 10.3389/fbioe.2023.1081072 (PMC10193151; doi:10.3389/fbioe.2023.1081072)
Supplement: Supplementary file 1 [file DataSheet1.pdf]

*Supplementary Material*

# Tailoring the HHx monomer content of P(HB-*co*-HHx) by flexible substrate compositions: scale-up from deep-well-plate to laboratory bioreactor cultivations

**Lara Santolin[†,1], Isabel Thiele[†,1], Peter Neubauer[1], Sebastian L. Riedel[*,1,2]**

[1]Technische Universität Berlin, Institute of Biotechnology, Chair of Bioprocess Engineering, Ackerstraße 76, 13355 Berlin, Germany

[2]Berliner Hochschule für Technik, Department VIII – Mechanical Engineering, Event Technology and Process Engineering, Laboratory of Environmental and Bioprocess Engineering, Seestr. 64, 13347 Berlin, Germany

[†] These authors contributed equally to this work and share first authorship

*** Correspondence:**
Sebastian L. Riedel
riedel@tu-berlin.de, sebastian.riedel@bht-berlin.de

## 1 Evaluation of the Impact of Gum Arabic as an Emulsifier

To ensure the same canola oil concentration in all replicate wells, the media containing canola oil was emulsified according to the material and methods section. In order to test whether gum arabic could be utilized as a substrate or inhibit growth, mineral salt medium without canola oil and emulsifier was incubated as a negative control for 72 h at 30°C and 225 rpm in 24-deep-well-plates with 3 mL working volume. MSM provided with only gum arabic did not lead significant growth (MSM + GA). A slightly higher CDW in comparison to the negative control is obtained as the gum arabic does not evaporate during lyophilization so that it remains in the sample. Evaluation of the influence of gum arabic compared to cultivations without emulsifier was done in shake flask cultivations with 5 g $L^{-1}$ canola oil as carbon source. Comparable CDWs of 5.38 ± 0.24 (MSM + GA + CO) and 5.74 ± 0.17 g $L^{-1}$

(control +) were reached. Results indicated that gum arabic was a suitable emulsifier that did neither inhibit growth nor was utilized as a carbon source (Figure S1).

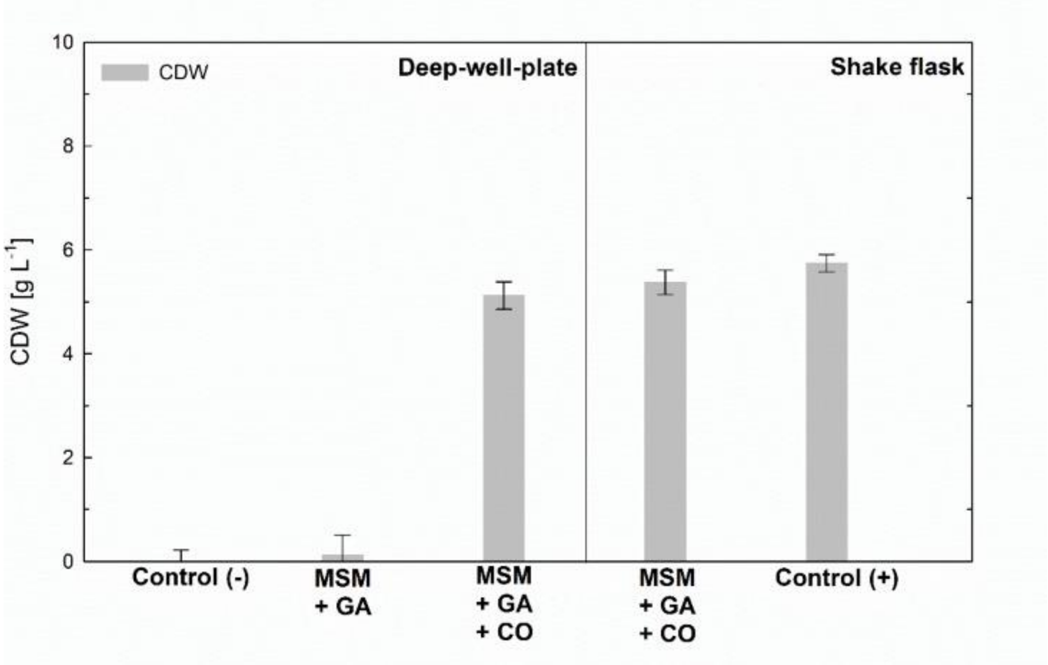


**Supplementary Figure 1.** Evaluation of the impact of gum arabic as an emulsifier in *R. eutropha* Re2058/pCB113 cultivations with canola oil as substrate. Cell dry weight (CDW; g $L^{-1}$) after 72 h at 30°C and 225 rpm (50 mm amplitude) of 3 mL deep-well-plate cultures and 50 mL shake flask cultures was determined. Deep-well-plate cultivations were performed either using mineral salt medium (negative control), mineral salt medium and gum arabic (MSM+GA) or with addition of canola oil (MSM+GA+CO) as carbon source (positive control). The latter condition was compared to the final CDW without the use of an emulsifier in shake flasks. Error bars indicate the standard deviation of five samples (deep-well-plates) or the standard deviation of biological duplicates (shake flasks).

## 2 Carbon Content and C/N ratios calculations

To calculate the carbon content of fructose ($CC_{Fructose}$,0.399 g $g^{-1}$) the molecular weight of fructose ($M_{Fructose}$) was divided by the number of carbon atoms in the molecule ($C_{Fructose}$) multiplied by the standard atomic weight of carbon ($A_r°(C)$) (1).

$$CC_{Fructose} = \frac{M_{Fructose}}{C_{Fructose} * A_r°(C)} \qquad (1)$$

The carbon content of canola oil was approximated at 0.775 g $g^{-1}$. For this, the fatty acid average composition in canola oil was withdrawn from (Orsavova et al., 2015) and an average molar mass of a fatty acid was calculated based on this composition. Following, the molar mass of an average triglyceride was calculated considering the molar mass of glycerol as well as the loss of one water molecule at each fatty acid bond. Finally, the averaged molar mass of a triglyceride was divided by the average number of carbon atoms present multiplied by its standard atomic weight (2).

$$CC_{Canola\ oil} = \frac{(3*M_{average\ FA}) + M_{glycerol} - M_{H2O}}{(3*C_{average\ FA} + C_{glycerol}) * A_r°(C)} \qquad (2)$$

To calculate the nitrogen content of urea ($NC_{Urea}$, 0.466 g $g^{-1}$) the molecular weight of urea ($M_{Urea}$) was divided by the number or nitrogen atoms in the molecule ($N_{Urea}$) multiplied by the standard atomic weight of nitrogen ($A_r°(N)$) (3).

$$NC_{Urea} = \frac{M_{Urea}}{N_{Urea} * A_r°(N)} \qquad (3)$$

The C/N (g $g^{-1}$) ratio was calculated as the total carbon (g) represented by the amount of fructose and canola oil in each mixture and dividing it by the total nitrogen (g) supplied as urea (4).

$$\frac{C}{N} = \frac{C_{Fructose} + C_{Canola\ oil}}{N_{Urea}} \qquad (4)$$

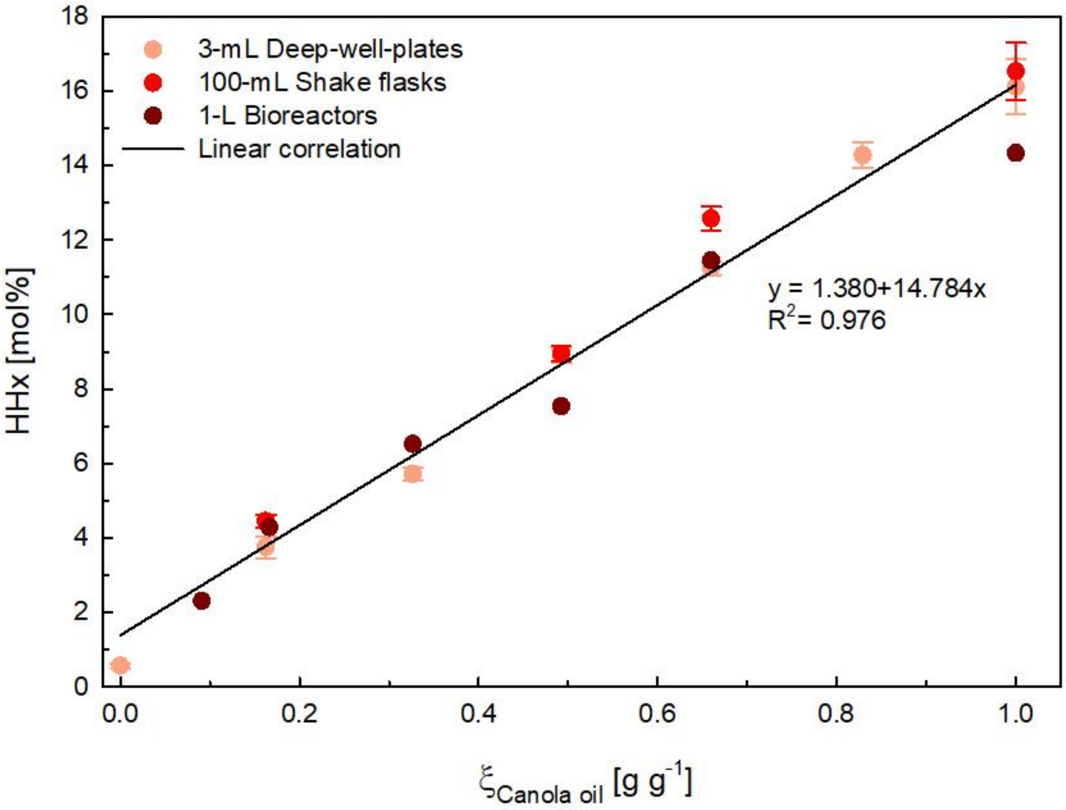


**Supplementary Figure 2.** Linear correlation between the canola oil ratio in fructose and canola oil mixtures and the HHx content of P(HB-*co*-HHx) obtained with *R. eutropha* Re2058/pCB113 cultivations with urea as nitrogen source. Results of 3-mL deep-well-plate cultivations with total carbon 5 g $L^{-1}$ (pink dots), 100-mL shake flask cultivations with total carbon 10 g $L^{-1}$ (red dots) and 1-L bioreactor cultivations with total carbon 10 g $L^{-1}$ (brown dots), all with a C/N ratio of 22 g $g^{-1}$ are shown. The carbon mass fraction of canola oil in each mixture ($\xi_{Canola\ oil}$; g $g^{-1}$) is plotted against the HHx molar content (HHx, mol%) obtained. The equation for the linear correlation as well as its $R^2$ value is shown. For deep-well-plates error bars represent standard deviation from duplicate measurements of pulled samples. For shake flasks cultivations error bars represent standard deviation between biological triplicates.

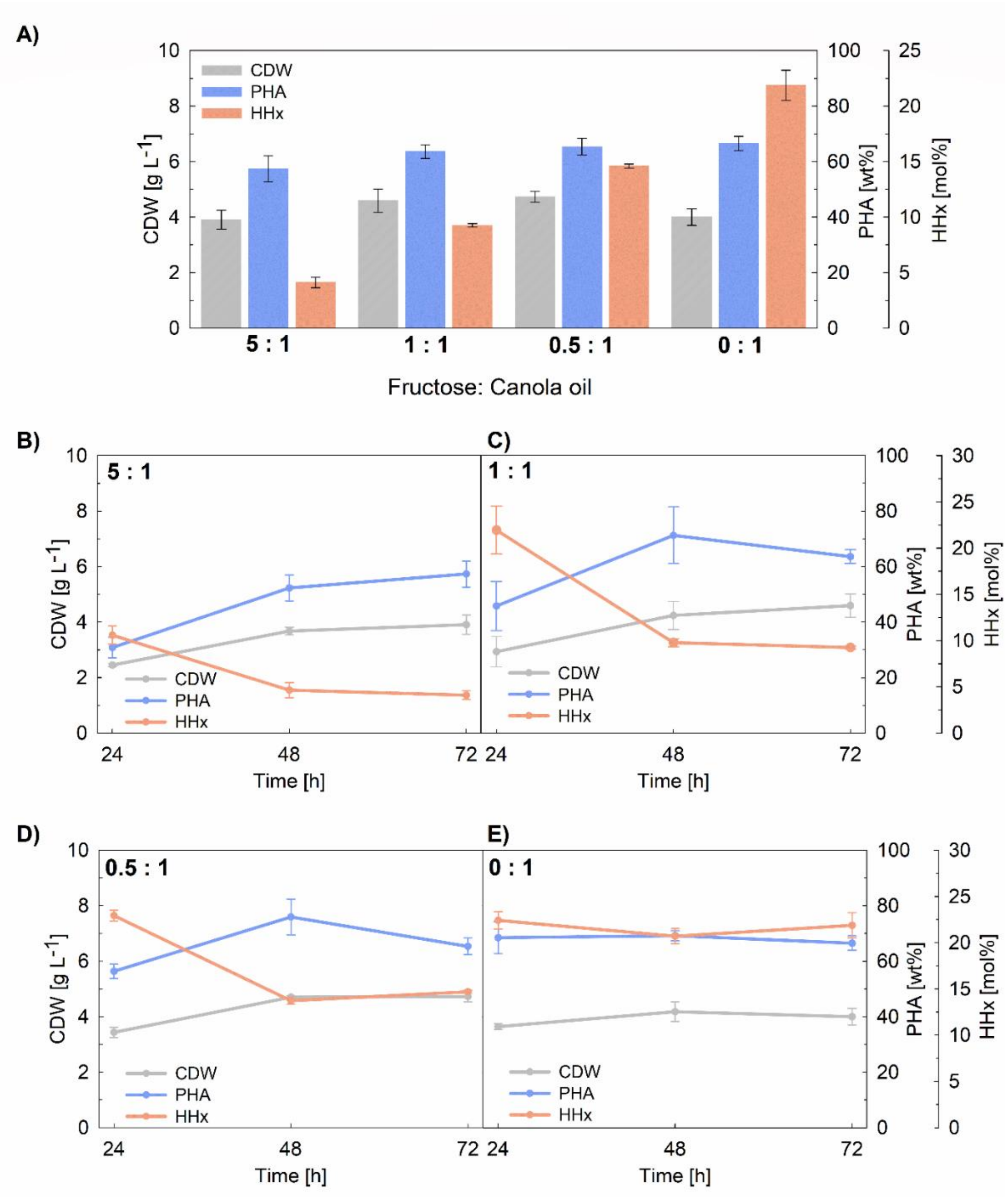


**Supplementary Figure 3.** 100-mL shake flask cultivations with *R. eutropha* Re2058/pCB113 using fructose and canola oil mixtures and urea as nitrogen source. Final yields of Cell dry weight (CDW; g $L^{-1}$), PHA content of CDW (PHA; wt%) and HHx content of PHA (HHx; mol%) after 72 h are shown in (A) as well as the samples every 24 h for each cultivation with 5 : 1 (B), 1 : 1 (C), 0.5 : 1 (D) and 0 : 1 (E) fructose to canola oil ratio and a final carbon content of 5 g $L^{-1}$ and a C/N ratio of 22 g $g^{-1}$. The carbon ratio of fructose to canola oil (g $g^{-1}$) is indicated for each mixture. Error bars indicate standard deviation from biological triplicates.

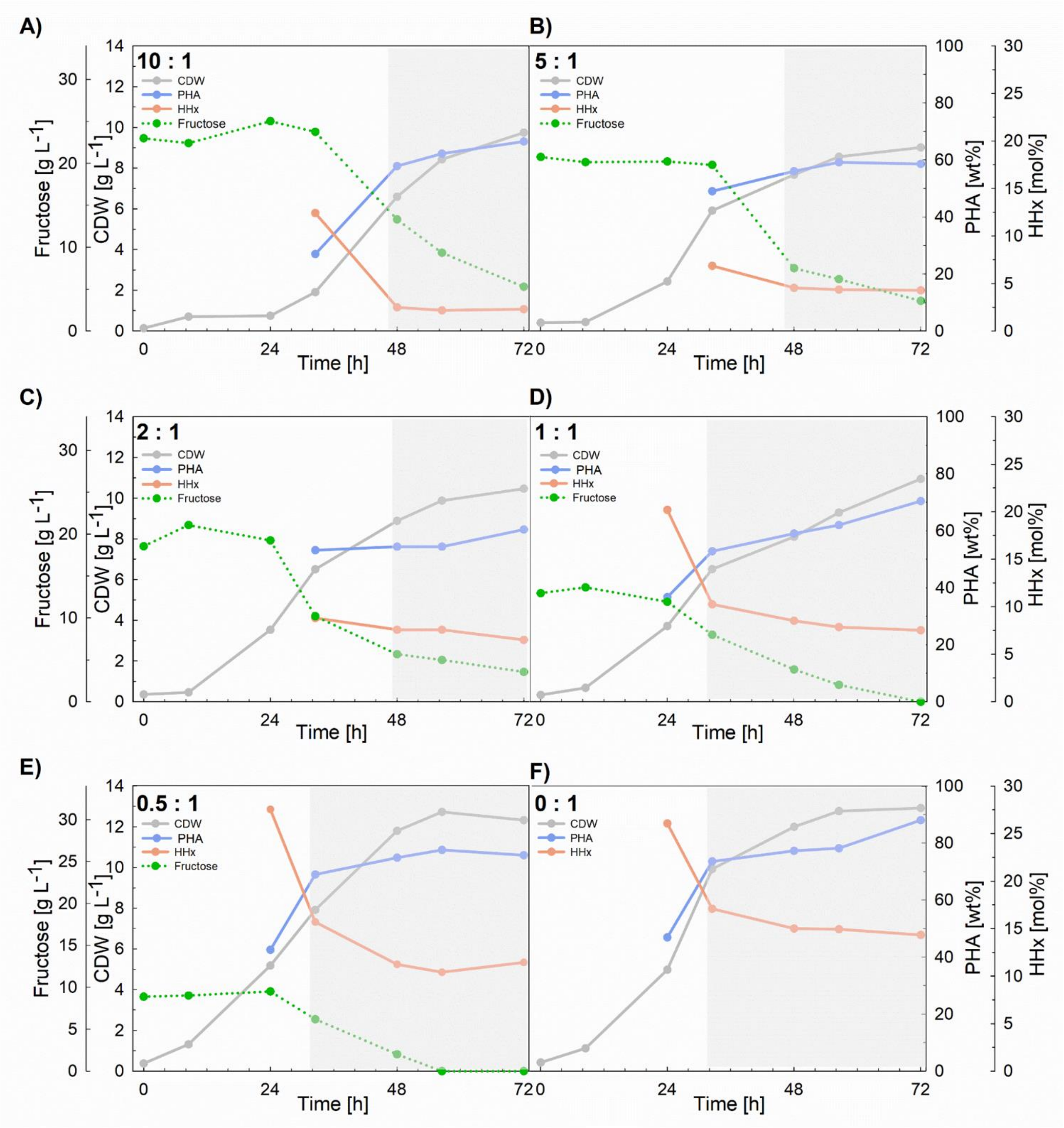


**Supplementary Figure 4.** 1-L bioreactor cultivations for tailor-made P(HB-*co*-HHx) production with *R. eutropha* Re2058/pCB113 using fructose and canola oil mixtures as carbon source and urea as nitrogen source. Cell dry weight (CDW; g $L^{-1}$), PHA content of CDW (PHA; wt%), HHx content of PHA (HHx; mol%) and fructose concentration (Fructose; g $L^{-1}$) values are shown over the course

of the cultivation for each mixture with a final carbon content of 10 g $L^{-1}$ and a C/N ratio of 22 g $g^{-1}$. The carbon ratio of fructose to canola oil (g $g^{-1}$) is indicated for each mixture. The shaded area indicates the polymer production phase after nitrogen limitation.

**Supplementary Table 1.** Molecular weight characteristics of samples from 1-L bioreactor cultivations with *R. eutropha* Re2058/pCB113 using mixtures of fructose and canola oil with a final carbon content of 10 g $L^{-1}$ and urea as nitrogen source with an applied C/N ratio of 22. Weight-average molecular weight ($M_w$; × $10^5$ Da), number-average molecular weight ($M_n$; × $10^5$ Da) and polydispersity index (Đ; -) are shown. Characteristics were determined from size exclusion chromatograms. Measurements represent means from duplicate measurements. ± are indicating minimum and maximum values.

| **Fructose : Canola oil [g $g^{-1}$]** | **Time [h]** | **$M_w$ × $10^5$ [Da]** | **$M_n$ × $10^5$ [Da]** | **Đ [-]** |
|---|---|---|---|---|
| **10 : 1** | 48 | 3.9 | 1.6 | 2.5 |
| | 56.5 | 3.8 | 1.6 | 2.4 |
| | 72 | 3.5 | 1.4 | 2.5 |
| **5 : 1** | 32.5 | 4.1 | 1.8 | 2.3 |
| | 48 | 4.3 | 1.8 | 2.4 |
| | 56.5 | 4.2 ± 0.0 | 1.7 ± 0.9 | 2.5 ± 0.1 |
| | 72 | 4.0 ± 0.1 | 1.6 ± 0.1 | 2.4 ± 0.0 |
| **2 : 1** | 32.5 | 3.1 | 1.4 | 2.3 |
| | 48 | 3.3 ± 0.0 | 1.4 ± 0.0 | 2.3 ± 0.0 |
| | 56.5 | 3.2 ± 0.0 | 1.4 ± 0.0 | 2.3 ± 0.0 |
| | 72 | 3.2 ± 0.0 | 1.3 ± 0.0 | 2.4 ± 0.0 |
| **1 : 1** | 32.5 | 3.2 | 1.5 | 2.1 |
| | 48 | 3.3 ± 0.0 | 1.4 ± 0.0 | 2.3 ± 0.0 |
| | 56.6 | 3.4 ± 0.0 | 1.5 ± 0.0 | 2.3 ± 0.0 |
| | 72 | 3.5 ± 0.0 | 1.5 ± 0.0 | 2.3 ± 0.0 |
| **0.5 : 1** | 48 | 3.6 ± 0.1 | 1.7 ± 0.0 | 2.2 ± 0.0 |
| | 56.5 | 3.5 ± 0.0 | 1.6 ± 0.0 | 2.2 ± 0.1 |
| | 72 | 3.6 ± 0.1 | 1.7 ± 0.1 | 2.2 ± 0.1 |
| **0 : 1** | 32.5 | 3.8 ± 0.1 | 1.8 ± 0.1 | 2.1 ± 0.0 |
| | 48 | 3.6 ± 0.1 | 1.7 ± 0.0 | 2.2 ± 0.0 |
| | 56.5 | 3.2 ± 0.1 | 1.4 ± 0.1 | 2.3 ± 0.0 |
| | 72 | 3.4 ± 0.1 | 1.5 ± 0.0 | 2.3 ± 0.0 |
